# The diagnosis and arthroscopic treatment of angioleiomyoma presenting loose body in the knee joint: two case reports

**DOI:** 10.1186/s12891-018-2087-6

**Published:** 2018-05-24

**Authors:** Chenxi Cao, Zhengming Cao, Guangyu Liu, Songyang Liu, Yanqi Ye, Tiezheng Sun

**Affiliations:** Arthritis Clinic and Research Center, Peking University, People’s Hospital, Beijing, 100044 People’s Republic of China

**Keywords:** Angioleiomyoma, Loose body, Intra-articular, Knee, Arthroscopy

## Abstract

**Background:**

Angioleiomyoma is a very rare benign solitary soft tissue neoplasm originating from smooth muscle layer of blood vessels. The tumor is usually located in the subcutis or the superficial fasciae, but less often in the deep fasciae, especially rare in the knee joint cavity. Diagnosis is frequently delayed or misdiagnosed as loose body or anterior knee pain because of its rare occurrence and poor awareness of physicians. Few studies have presented intra-articular angioleiomyoma and such cases become rarer and more difficult to diagnose when it presents as loose body.

**Case presentation:**

Two patients, a middle-aged man and an old woman, presented to our outpatient clinic with persistent anterior knee pain and both of them suffered from a solitary mass in the right knee that had slowly enlarged. One of two patients showed negative in the routine radiographic imaging and the other showed a “loose body” beside the lateral femoral condyle in the knee. MRI showed both a well-demarcated intra-articular mass of isointense signal to muscle on T1-weighted images and heterogeneous intensity on T2-weighted images. Their tumors were excised under arthroscopy finally, with the pathological results revealed vascular leiomyomas. They both recovered well with pain free after operation and no signs of recurrence were seen at the 7-year follow-up.

**Conclusions:**

This case report illustrates the atypical locations of angioleiomyoma in the knee joint should arouse our attention and be included in the differential diagnosis of nodular lesions mimicking loose bodies.

## Background

Angioleiomyoma, also known as vascular leiomyoma, is a rare benign soft tissue tumor of smooth muscle origin arising from the muscular layer of vessel walls [1]. The most common presentation is a painful solid subcutaneous swelling. It usually occurs in subcutaneous fat and fascia with the favored site being the extremities, particularly lower limbs. The peak incidence is in the fourth to the sixth decades of life, with a female preponderance [2]. Previous literatures showed it can be found anywhere in the body such as the uterus, crania, atrium, hard palate, nasal septum [3–6]. Although angioleiomyoma commonly affects the lower extremities, knee joint is rarely located, especially the intra-articular angioleiomyoma which is presented as loose body. As far as we know, only a minority of individual cases have been reported [7–9]. Herein, we presented two cases suffered from a solitary and mobile mass in the knee cavity, which was demonstrated as angioleiomyoma by the pathological examination after complete resection under arthroscopy.

## Case presentation

### Case 1

**Fig. 1:**
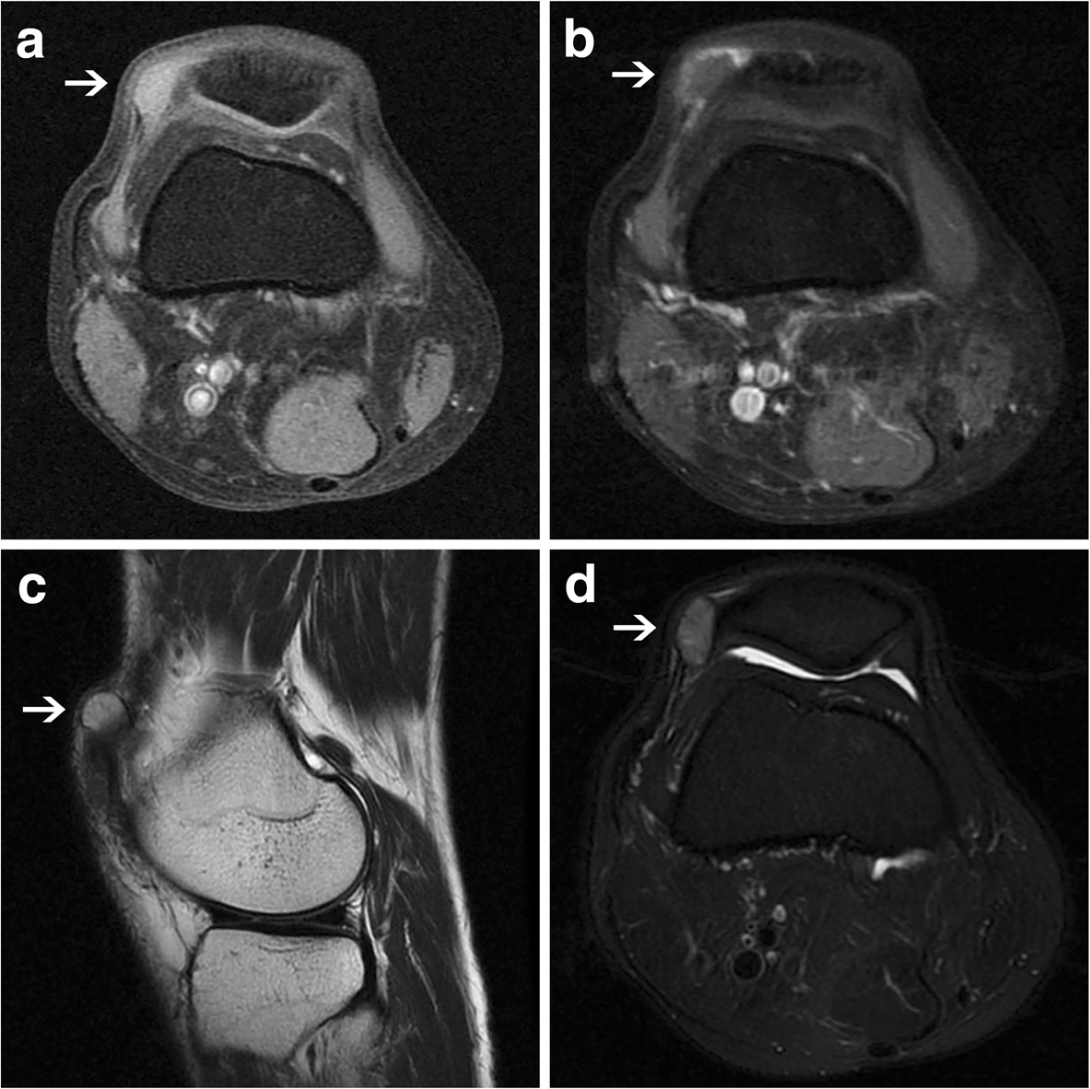
MRI showed the mass presented isointense signal to muscle on STIR T1 sequence (**a**), and was heterogeneously distinctly strengthened on enhanced STIR T1 sequence (**b**). The MRI also showed slightly hyperintense signal on T2WI (**c**) and STIR T2 sequence (**d**)

**Fig. 2:**
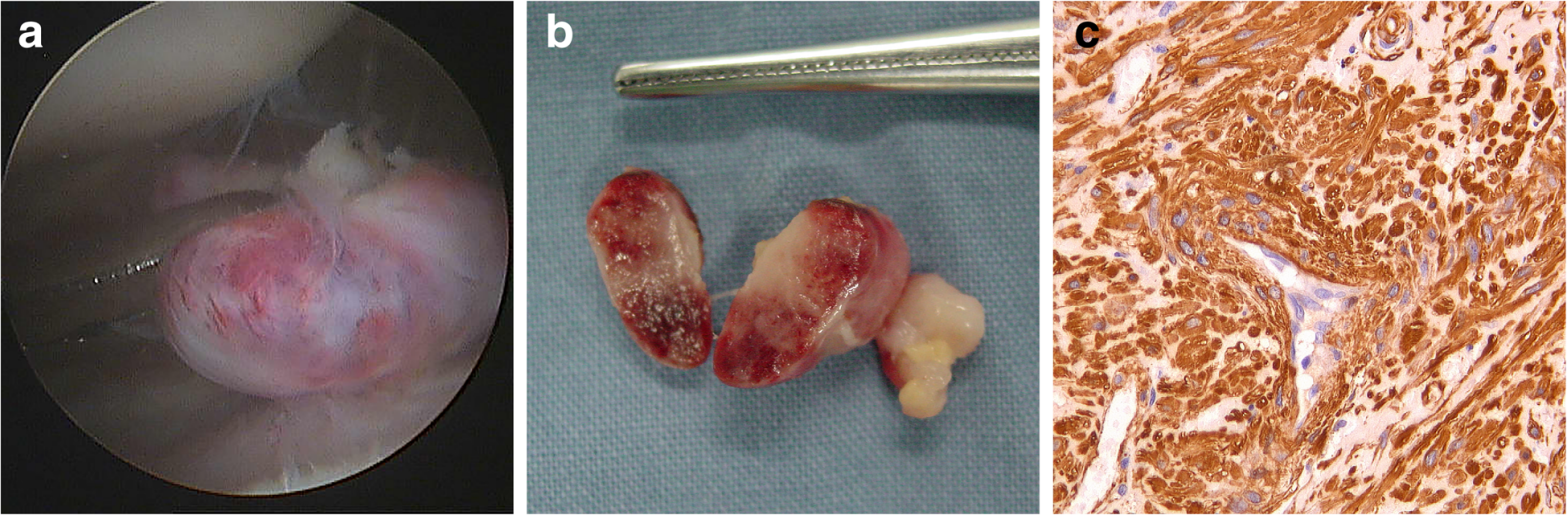
A red, oval mass was showed at arthroscopy in the right knee (**a**). Grossly, the tumor was red, firm, approximately 1.5 cm × 1.0 cm in size, covered with intact fibrous capsule, with inhomogeneous dark red color on cut surface (**b**). Immunohistochemical staining (**c**) showed the mass composed of closely crossed smooth muscle bundles surrounding split-like vascular channels with full fibrous capsule and SMA was significantly positive (200×)

A healthy 41-year-old man presented with a mass in his right knee that had slowly enlarged over the past 15 years. Physical examination revealed a solid, glossy and mobile swelling beside the lateral upper pole of the right patella which was approximately 2 cm in the largest dimension and was mildly tender to palpation. Routine radiographic imaging was negative, magnetic resonance imaging (MRI) demonstrated a well-demarcated oval lesion measuring 1.5 cm in diameter within the soft tissue abutting the lateral upper pole of the patella, which presented isointense signal to muscle on STIR T1 sequence and slightly hyperintense signal on T2 weighted images (T2WI) and STIR T2 sequence, and the edge of the mass was heterogeneously strengthened on enhanced T1 weighted images (Fig. 1). MRI considered it was a loose body or benign lesion firstly and hemangioma was not excluded. The mass was easily visualized after lateral release along the patella during arthroscopy and was completely excised (Fig. 2a). Grossly, a macroscopic examination revealed an oval, smooth faced and soft excised mass which was dark red in color and measured 1.5 cm × 1.0 cm. When being cut into halves, it was tough and covered with intact fibrous capsule (Fig. 2b). The following pathological results confirmed it to be an angioleiomyoma and the immunohistochemical staining showed smooth muscle actin (SMA) (++), (Fig. 2c). The patient experienced a complete resolution of his symptoms postoperatively and did not recur within an 8-year follow-up.

### Case 2

**Fig. 3:**
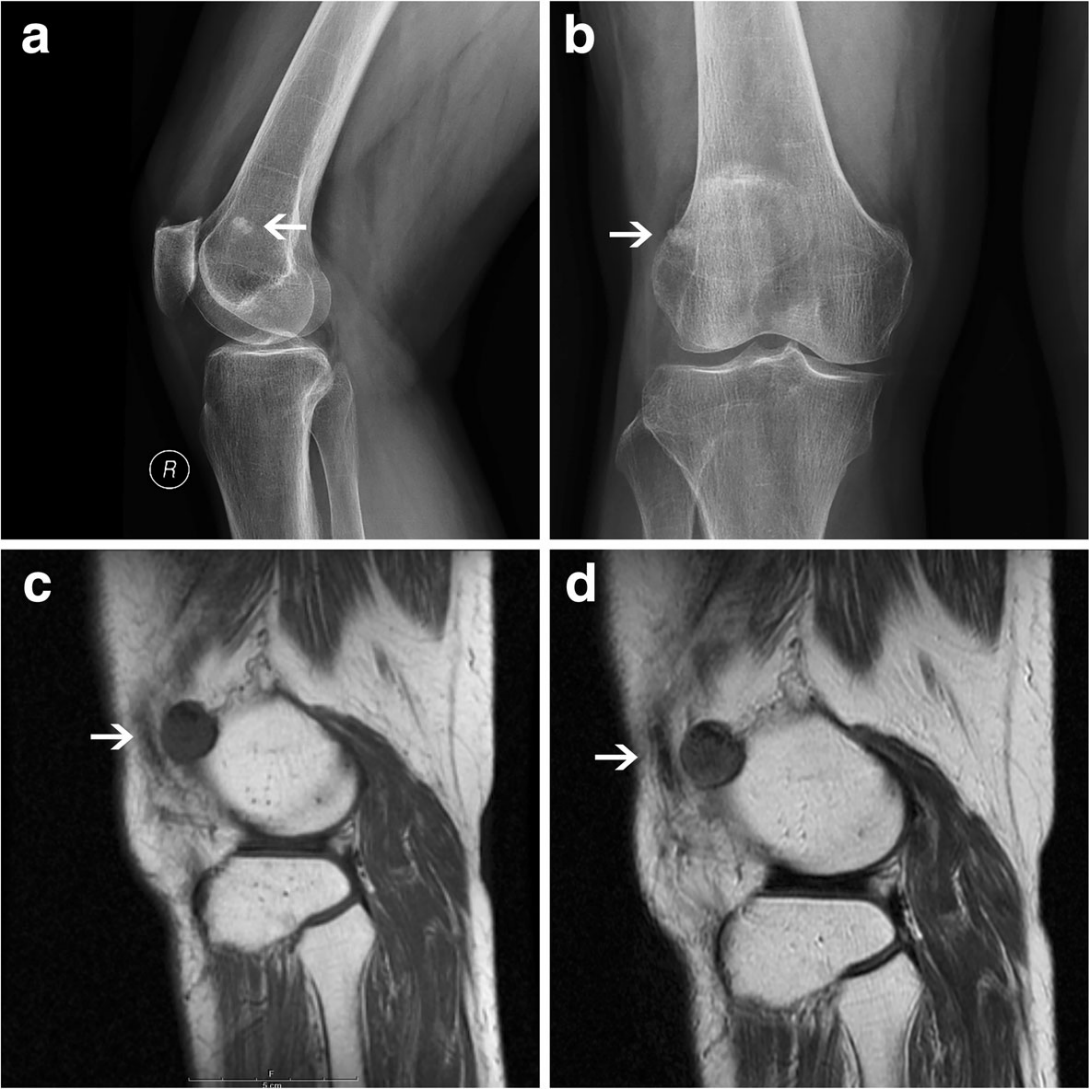
Radiograph plain film showed a “loose body” beside the lateral femoral condyle in the right knee (**a, b**). MRI showed the signal of the nodule was not uniform and isointense to muscle on T1WI (**c**) and T2WI (**d**)

**Fig. 4:**
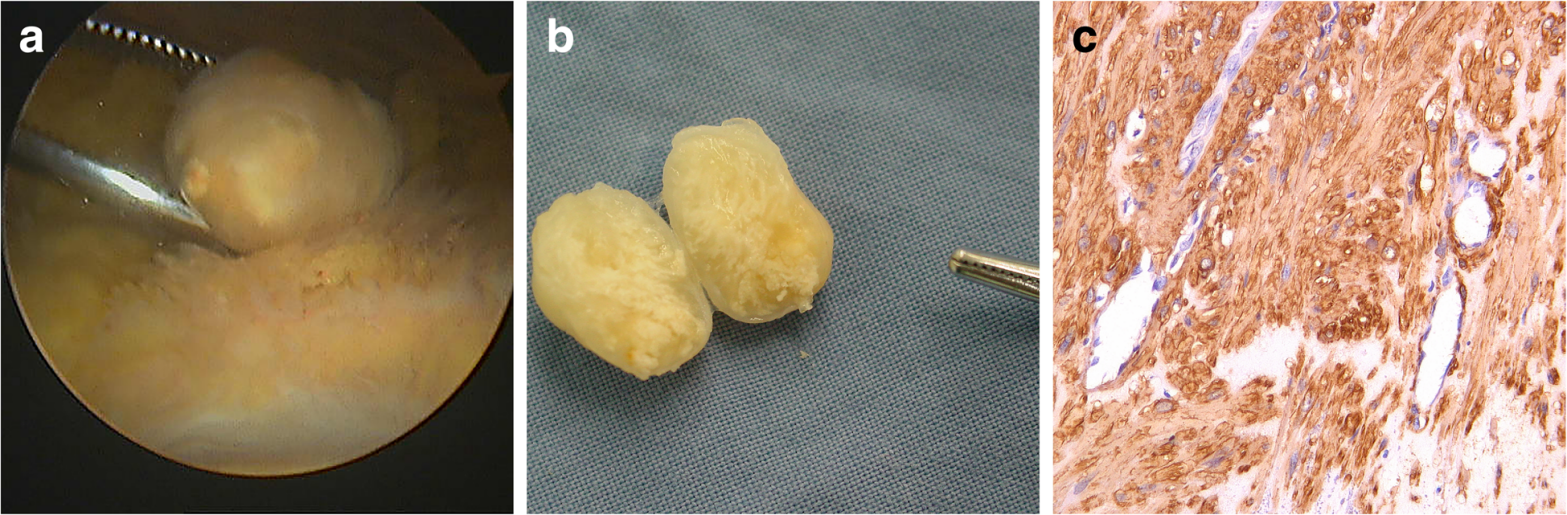
The arthroscopy showed a yellow, firm and spherical mass in the knee (**a**). Macroscopically, the lesion was yellow, about 1.5 cm in diameter, covered with full envelope, uneven yellow in the section and diffusely calcified (**b**). Immunohistochemical staining (**c**) showed SMA was significantly positive and diffuse calcification and ossification could be seen (200×)

A 72-year-old lady was referred to our hospital with a slowly growing, painful mass located on the right knee that had been presented for over 20 years. Clinical examination disclosed a tender, movable and smooth mass about 2 cm in diameter. X-ray showed a “loose body” beside the lateral femoral condyle in the knee (Fig. 3). MRI demonstrated that the nodule was spherical, 1.8 cm × 1.6 cm in size, well-defined and calcified. The signal of the nodule was not uniform and isointense to muscle on T1WI and T2WI (Fig. 3). MRI considered a benign lesion like a loose body, which was probable synovial origin. At arthroscopy, the mass was attached to the lateral femoral condyle (Fig. 4a) and was wholly removed, which was yellow, about 1.5 cm in diameter, covered with full envelope, firm, uneven yellow in the section and diffusely calcified (Fig. 4b). The histological examination was consistent with an angioleiomyoma with calcification, and the diagnosis was confirmed by immunohistochemistry (Fig. 4c). The patient recovered well with pain free after operation and no signs of recurrence were seen at the 7-year follow-up.

## Discussion and conclusions

Angioleiomyoma can occur anywhere in the body and is most often seen in the extremities, particularly the lower limbs [8], but very rare in the knee joint. Al-Jabri et al. reported a case of an angioleiomyoma presented with recurrent pain and a soft tissue swelling at the posteromedial aspect of right knee [7]. Murty AN et al. reported a case of solitary angioleiomyoma arising in the infra-patellar fat pad and presenting with anterior knee pain [10]. We reported two cases of angioleiomyomas presenting loose bodies in the knee joint, and the case of angioleiomyoma with calcification in the knee should be the first report in the literatures.

Intra-articular tumors are very unusual and physicians are apt to misdiagnose conditions like meniscus tear or arthritis [11]. To avoid making a misdiagnosis, a high index of suspicion is required. Mazen Hamoui et al. suggested that angioleiomyoma should be included in the differential diagnosis of nodular lesions mimicking loose body, such as lipomas, inclusion cysts, ganglion, pigmented villonodular synovitis (PVNS), fibroma, nodular synovitis, hemangioma, synovial sarcoma, myopericytoma, leiomyosarcomas, glomus tumor, etc. [12]. Usually an angioleiomyoma presents as a painful, solitary, small, slow-growing and mobile lesion, the majority are oval and less than 2 cm in size [13]. These findings are consistent with our patients. Clinical onset of the angioleiomyoma often reflects the extension of the lesions, but it does not have typical clinical manifestations. The most characteristic complaints are pain and tenderness. Pain is often paroxysmal in nature and is temperature sensitive and provoked by touch [14], it may be due to smooth muscle contraction, irritation of the involved nerve, or ischemia caused by blood vessel spasms.

There are no specific imaging techniques for vascular leiomyomas. Radiograph plain film has little value in diagnosis except for the calcification of the tumors. MRI is helpful for diagnosis but still not specific. Angioleiomyoma is difficult to be diagnosed before surgery and it should be considered a possible diagnosis when a well-demarcated mass of isointense signal to muscle on T1WI, heterogeneous high signal intensity on T2WI, heterogeneous strong enhancement, and an adjacent tortuous vascular structure is seen in the extremities [15]. Alta YT Lai et al. retrospected and analyzed the MRI features of 37 cases of vascular leiomyomas, the results showed that on T1WI, most of the cases showed isointense or slightly high signal intensity compared to adjacent muscle, a few cases showed heterogeneous or low signal intensity; and on T2WI, all the cases indicated high signal intensity; their review results also demonstrated that on gadolinium enhanced T1WI, about 42% were homogeneous enhancement, 39% were heterogeneous enhancement and only one case showed peripheral enhancement [16]. The case 1 in the current report had the similar MRI characteristics as above which showed isointense compared to adjacent muscle on TIWI and peripheral enhancement on gadolinium enhanced T1WI. However, the presence of fat, haemorrhage, hyalinization and calcification within the mass did result in a notable variation of the MRI characteristics [17]. Aditya V. Maheshwari et al. reported it was mildly heterogeneous on fat saturated T2WI and isointense to muscle on T2WI and STIR images in a case of calcified angiomyomas of the foot [18]. The cases 2 in the current report showed the isointense to muscle on T2WI according to calcification.

As the symptoms were nonspecific, the final diagnosis was made only after arthroscopic removal. Macroscopically, angioleiomyomas are oval, covered with full fibrous capsule, firm, yellow or red on the cut surface, and may be calcified or ossified. Microscopically, the lesions are composed of varying amounts of smooth muscle bundles surrounding vascular channels contained within a thin fibrous capsule. Immunohistochemically, Matsuyama et al. analyzed 122 angioleiomyoma cases and showed tumor cells strongly reactive to SMA, muscle-actin monoclonal antibody (HHF35), and actin associated antibody (CALP) in all of cases. Desmin was diffusely stained in 63.1%, partially positive in 19.7%, and not stained in 17.2% [19]. Calcification or ossification in angioleiomyoma is very rare. In our two cases, SMA is strongly and diffusely positive, and calcification can only be seen in one case.

Many reports have proved successful arthroscopic resection of benign intra-articular lesions of the knee, such as localized PVNS, synovial chondromatosis, synovial angioma, osteochondroma, lipoma arborescens and synovial lipoma [20–22]. Complete excision with arthroscopy was considered adequate and there were no cases of recurrence after up to 7 years of follow-up in the current two cases of angioleiomyomas. However, many patients with primary bone tumors and soft tissue tumors presenting around the knee joint have been misdiagnosed as sports injuries [11, 23], which may lead to mistakes in selection of therapeutic approach. Arthroscopy is a minimally invasive procedure for diagnosis and treatment of knee joint disease, but still have adverse consequences in the presence of an unsuspected neoplasm. In some cases, it is possible to change from a conservative tumoral resection to radical surgery or amputation [24].

As this case report illustrated, the atypical locations of angioleiomyoma in the knee joint should arouse our attention and be included in the differential diagnosis of nodular lesions mimicking loose bodies.

## Abbreviations

CALP: Actin associated antibody
HHF35: Muscle-actin monoclonal antibody
MRI: Magnetic resonance imaging
PVNS: Pigmented villonodular synovitis
SMA: Smooth muscle actin
T1WI: T1 weighted images
T2WI: T2 weighted images
